# Selective retinal neuron loss and impaired neurovascular support underlie myopic retinopathy in RPE-specific Lrp2-deficient mice

**DOI:** 10.3389/fncel.2026.1856140

**Published:** 2026-07-30

**Authors:** Jinyan Qi, Xuexue Cui, Caijiao Yi, Jian Liu, Wen Deng, Xiang-Ling Yuan, Yuanyuan Hu, Tong Wang, Heping Xu

**Affiliations:** 1 Aier Eye Institute, Changsha Aier Eye Hospital, Changsha, Hunan, China; 2 Beijing AIER-Intech Eye Hospital, Beijing, China; 3 Aier Academy of Ophthalmology, Central South University, Changsha, Hunan, China

**Keywords:** axial elongation, microglia, microvascular degeneraton, neurodegeneration, pathologic myopia

## Abstract

Pathologic myopia is a major cause of irreversible visual impairment worldwide and is characterized by excessive axial elongation accompanied by progressive retinal degeneration. Whether vision loss results primarily from passive retinal stretching or selective neurodegeneration remains unclear, hindering the development of effective neuroprotective and regenerative therapies. Here, we investigated retinal neuronal, vascular, and glial alterations in retinal pigment epithelium (RPE)-specific Lrp2 knockout (Best1-Cre/Lrp2^fl/fl^ conditional knockout, CKO) model of pathologic myopia. The CKO mice were examined longitudinally using multimodal ocular imaging, electroretinography, optokinetic testing, fluorescein angiography, and quantitative immunohistochemistry analysis. CKO phenotype^+^ mice developed early-onset, progressive axial elongation and high myopia, accompanied by fundus features closely resembling human pathologic myopia, including peripapillary and patchy chorioretinal atrophy. Retinal function was markedly impaired, with significant reductions in scotopic a-, b-, and c-wave amplitudes. Although axial elongation resulted in a 1.98-fold increase in retinal surface area and a 55.95% reduction in retinal thickness, quantitative correction for retinal expansion revealed selective neuronal loss rather than uniform retinal degeneration. Total numbers of rods, cones, horizontal cells, and GABAergic amacrine cells were reduced by 22, 40, 30, and 57%, respectively, together with a 66% loss of photoreceptor synaptic ribbons. In contrast, retinal ganglion cells and bipolar cells exhibited reduced density but preserved absolute cell numbers. These neuronal changes were accompanied by retinal and choroidal microvascular degeneration, Müller gliosis, microglial activation and subretinal accumulation, and RPE dysmorphology. Our findings demonstrate that axial elongation induces neuron subtype–specific degeneration rather than generalized retinal thinning. Our study identifies photoreceptors, horizontal cells, and inhibitory amacrine cells as particularly vulnerable populations and implicates impaired RPE support, neurovascular dysfunction, and chronic glial activation as key mechanisms driving myopic retinopathy. This study provides a mechanistic framework for developing targeted neuroprotective and regeneration-based therapies for pathologic myopia.

## 1 Introduction

Myopia has emerged as a major global public health challenge, while pathological myopia representing a leading cause of irreversible visual impairment worldwide (Holden et al., 2016), affecting 3% of the global population (Wong et al., 2014) and 12–27% of Asian populations (Hsu et al., 2004; Iwase et al., 2006; Xu et al., 2006). Beyond refractive error, pathological myopia is characterized by excessive axial elongation of the eyeball accompanied by progressive retinal thinning, retinal and choroidal vascular degeneration, and neuronal dysfunction (Ohno-Matsui et al., 2021). These changes culminate in myopic retinopathy, a degenerative condition that shares features with other chronic neurodegenerative diseases but the cellular mechanisms remain poorly understood.

Clinically, myopic retinopathy is marked by macular atrophy, lacquer cracks, Fuchs spots, patchy chorioretinal atrophy or choroidal neovascularization (mCNV), and myopic traction maculopathy (Sayanagi et al., 2022; Ohno-Matsui, 2025), and increasing susceptible to secondary complications such as macular degeneration and glaucoma (Ng et al., 2023; Sun et al., 2023). Although retinal thinning and reduced neuronal density are well documented in highly myopic eyes, it remains unclear whether neuronal changes primarily reflect passive tissue stretching over an enlarged retinal surface or active, cell type–specific neurodegeneration. Dissecting these possibilities is essential for understanding vision loss in myopia and for developing effective neuroprotective strategies for myopia.

The retinal pigment epithelium (RPE) plays a central role in maintaining retinal homeostasis by supporting photoreceptor metabolism, regulating ion and fluid transport, controlling immune privilege (Strauss, 2005; Nussenblatt et al., 2014; Chen et al., 2016), and mediating bidirectional signaling between the retina and choroid. Disruption of RPE function has been implicated in both retinal degeneration and abnormal eye growth (Rymer and Wildsoet, 2005; Guymer and Campbell, 2023). Low-density lipoprotein receptor–related protein 2 (LRP2, also known as megalin) is a multifunctional endocytic receptor highly expressed in the RPE (Delaunay et al., 2025), where it regulates the uptake and signaling of multiple ligands, including morphogens and growth factors involved in ocular development (Yuan et al., 2023; Delaunay et al., 2025). LRP2 mutation is linked to Donnai-Barrow Syndrome, including craniofacial anomalies, sensorineural hearing loss, renal tubular dysfunction, and distinctive ocular features. Mice with Lrp2 deficiency develop enlarged eyeballs and retinal abnormalities (Storm et al., 2014, 2019; Cases et al., 2015), offering the opportunity to investigate the cellular mechanisms of myopic retinal degeneration.

In this study, we investigated the eye growth, retinal structure, neuronal composition, vasculature, and immune activation in CKO phenotype^+^ mice. We show that RPE-specific Lrp2 deficiency induces early-onset and progressive ocular enlargement accompanied by retinal thinning, vascular degeneration, gliosis, and microglial activation, closely resembling pathological myopia. Importantly, we demonstrate that axial elongation is associated with selective degeneration of photoreceptors, horizontal cells, and GABAergic amacrine cells, whereas other retinal neurons exhibit reduced density without a loss of absolute cell number. These findings identify neuron subtype–specific vulnerability as a defining feature of myopic retinopathy and highlight RPE and neurovascular unit as critical regulators of retinal integrity during excessive eye growth.

## 2 Materials and methods

### 2.1 Animals

Best1-Cre/Lrp2^fl/fl^ conditional knockout (CKO) mice were generated by crossing Best1-Cre mice (Jackson Laboratory, C57BL/6 J background) with Lrp2^fl/fl^ mice (Cyagen Bioscience, C57BL/6 J background). Cre-negative littermates (Lrp2^fl/fl^) were used as wild-type (WT) controls. Genotyping was performed by PCR using pre-weaning tail biopsies. Tail tissues were lysed in 10 μL of YSYBuffer DNA lysis buffer, and the Best1-Cre transgene and floxed Lrp2 allele were detected by PCR. Primer sequences are listed in Supplementary file 1 Table S1. The animal study was approved by the Ethics Committee of Aier Eye Institute (Ref: AEI20230012) and all procedures complied with the ARVO Statement for the Use of Animals in Ophthalmic and Vision Research.

### 2.2 Measurement of refractive error and ocular biometrics

Mice were anesthetized via intraperitoneal injection of tiletamine–zolazepam (40 mg/kg) (Zoletil® 50; Virbac, Carros-Cedex, France; 25 mg/mL tiletamine and 25 mg/mL zolazepam) and xylazine (15 mg/kg) (Shengda, Dunhua, China), followed by topical application of 0.5% tropicamide (Santen Pharmaceutical, Japan) for pupil dilation. Following completion of the experimental procedures, mice were euthanized by cervical dislocation while under deep anesthesia. During image acquisition, the cornea was kept moist with carboxymethyl cellulose sodium eye drops (1%; Allergan, Ireland), while autofocus and refractive compensation optimized image quality. Ocular biometric parameters, including axial length (AL), vitreous chamber depth (VCD), central corneal thickness (CCT), lens thickness (LT), and anterior chamber depth (ACD), were measured using SS-OCT (VG200; Intalight, China).

Spherical equivalent refractive error was determined under dim light conditions by two blinded optometrists using streak retinoscopy with trial lenses in hand-held, lightly restrained mice after anesthesia and pupil dilation. Measurements were obtained in triplicate for each eye.

### 2.3 Intraocular pressure (IOP) measurement

IOP was measured noninvasively using an Icare TonoLab tonometer (Tiolat i-care, Finland). Mice were gently restrained on a soft towel and remained calm during measurement. Readings were obtained within 3 min after anesthesia induction, and the reported IOP value was the mean of six consecutive measurements per eye.

### 2.4 Fundus imaging and retinal thickness analysis

Following anesthesia and pupil dilation, fundus imaging and retinal thickness measurements were performed using a Phoenix Micron IV SD-OCT system (Phoenix Research Laboratories, Pleasanton, CA, USA). Retinal thickness was measured at 500 μm from the optic disc along the dorsal–ventral and nasal–temporal meridians. Total, inner, and outer retinal thicknesses were defined as the distances from the retinal nerve fiber layer (RNFL) to the RPE, from the RNFL to the inner nuclear layer (INL), and from the outer plexiform layer (OPL) to the RPE, respectively. Measurements from each animal were averaged for statistical analysis.

### 2.5 Visual function assessment

Scotopic electroretinography (ERG): Scotopic ERG was recorded using a standard Ganzfeld stimulator (LKC Technologies, Gaithersburg, MD, USA). After overnight dark adaptation, mice were anesthetized and pupils dilated. The amplitudes and implicit times of a-wave, b-wave, oscillatory potentials (OPs), and c-wave were analyzed. C-wave amplitude was defined as the maximal positive wave amplitude following the b-wave.

Optokinetic reflex (OKR): visual acuity was assessed using the OptoDrum system (StriaTech, Germany) as previously described (Zeng et al., 2022). The spatial frequency threshold (cycles/degree, c/d) was determined at 99.78% contrast using a staircase protocol. The highest spatial frequency that elicited a reliable tracking response was recorded as the visual acuity threshold.

### 2.6 Ocular blood flow imaging

OCTA images were acquired using the VG200 SS-OCTA system (VG200; Intalight, China) equipped with a small-animal fundus imaging module and mouse eye lens. During image acquisition, the cornea was kept moist with carboxymethyl cellulose sodium eye drops (1%; Allergan, Ireland), while autofocus and refractive compensation optimized image quality. All scans were centered on the optic nerve head (ONH), and only images with a signal strength of 9/10 or higher were included in the analysis.

Retinal layers were automatically segmented using the built-in van Gogh software and manually corrected, when necessary, especially in CKO phenotype^+^ mice. En face OCTA images were further segmented into the superficial vascular plexus (SVP), intermediate capillary plexus (ICP), and deep capillary plexus (DCP). The SVP was defined from the retinal nerve fiber layer (RNFL) to the lower one-third of the ganglion cell layer plus inner plexiform layer (GCL + IPL) complex, the ICP from the lower one-third of the GCL + IPL complex to the middle of the inner nuclear layer (INL), and the DCP from the middle of the INL to 25 μm below the INL–outer plexiform layer (OPL) junction. The choroidal layer was defined as the region from Bruch’s membrane (BM +0 μm) to the choroid–sclera interface. Projection artifacts were removed using the built-in AI-based correction algorithm. Vessel density and vessel length density were automatically measured within the 1–3 mm peripapillary annulus centered on the ONH as % of retinal scanning volume. In CKO phenotype^+^ mice, RPE degeneration was common and was not used as an exclusion criterion because reduced RPE pigmentation may improve visualization of choroidal flow signals.

### 2.7 Fundus fluorescein angiography (FFA)

Fundus fluorescein angiography was conducted using the Phoenix Micron IV system (Phoenix Research Laboratories). Briefly, anesthetized mice with dilated pupils received sodium fluorescein (1%, 5 μL/g body weight) injection subcutaneously into the posterior hip region within 2 s. Images were captured at 0, 1, 2, 5, and 10 s post-injection. Fluorescence intensity and leakage area were quantified in predefined retinal regions using ImageJ with background subtraction and consistent threshold settings. The optic disc and artifacts were excluded, and analyses were performed in a blinded manner.

### 2.8 Immunofluorescence

Eyes were fixed in 4% paraformaldehyde (PFA) for 4 h, then processed for cryosections or retinal and RPE–choroidal flatmount staining. Cryosections were blocked in 5% goat serum with 2% BSA, permeabilized with 0.1% Triton X-100 for 1 h at room temperature, incubated with primary antibodies overnight at 4 °C, followed by secondary antibodies for 1.5 h and DAPI counterstaining.

The flatmount samples were blocked in 10% goat serum, 2% BSA, and 1% Triton X-100 for 2 h at room temperature, then incubated with primary antibodies overnight at 4 °C and secondary antibodies for 2 h at room temperature. Detailed antibody information is provided in Supplementary file 1 Table S2.

### 2.9 Retinal cell quantification

Retinal length was defined as the linear distance from the optic nerve head to the ciliary body and was divided into three equal regions: posterior, equatorial, and peripheral. In each region, cell density was calculated as the number of marker-positive cells per 100 μm of retinal length. The total cell density was calculated as the average of the three regional densities. The estimated cell number in each region was calculated as: cell number = cell density × regional retinal length. The total estimated cell number was obtained by summing the corrected cell numbers from the posterior, equatorial, and peripheral regions. The raw data were presented in Supplementary file 2.

Rod photoreceptor numbers were estimated by subtracting cone counts from total outer nuclear layer (ONL) nuclei. Synaptic structures were quantified using ImageJ software, while horizontal, bipolar, amacrine, and ganglion cells were manually counted. All analyses were independently performed by two blinded investigators.

## 3 Statistical analysis

Data are presented as mean ± standard deviation (SD). For image-based quantifications, multiple images or retinal regions from the same animal were averaged to obtain a single value for animal. Images were analyzed by two independent researchers, one of whom was blinded to the sample identity. Each animal was considered an independent biological unit. Group comparisons were performed using one-way or two-way ANOVA, corrected for multiple comparisons using Bonferroni *post hoc* tests. A *p* value <0.05 was considered statistically significant.

## 4 Results

### 4.1 Refraction and ocular parameters in CKO mice

**Figure 1:**
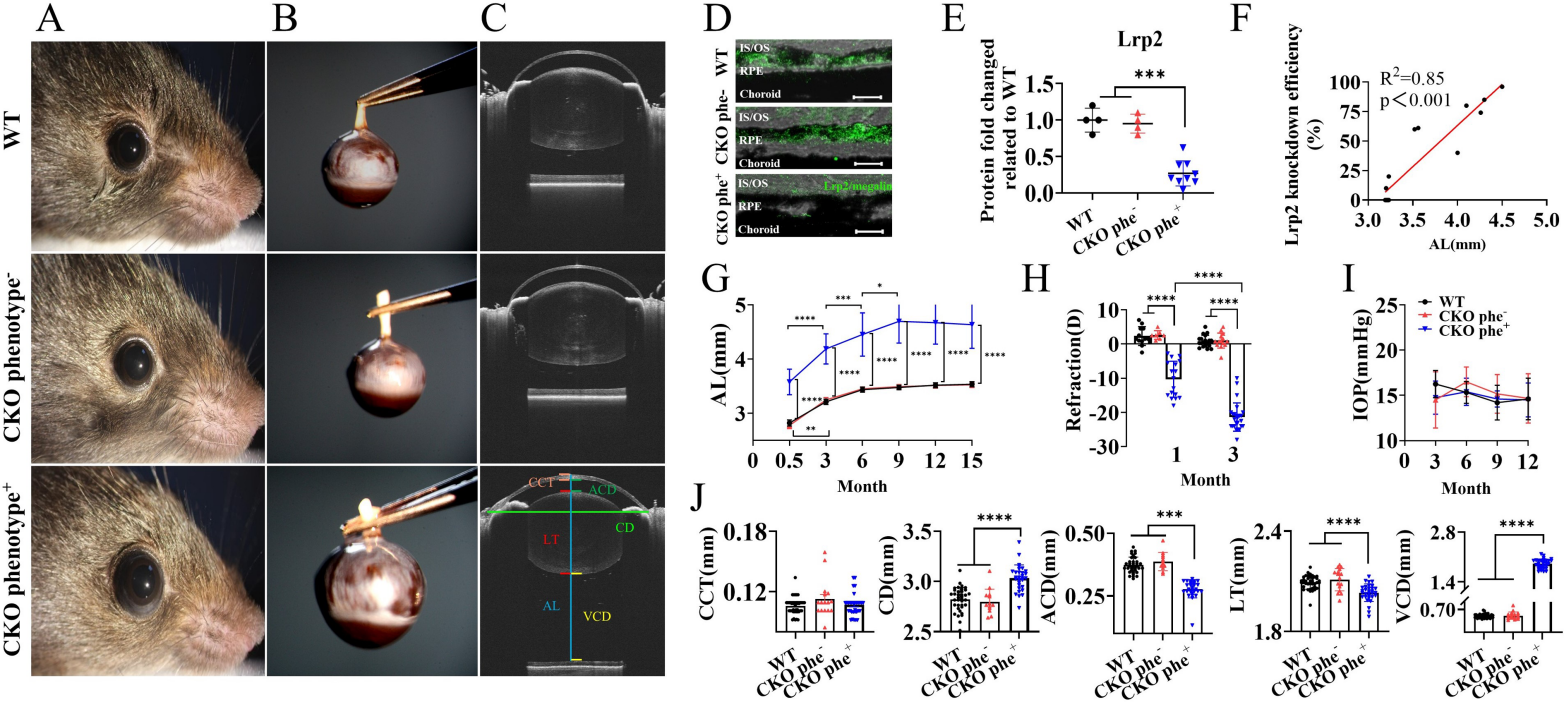
Ocular phenotypes in CKO mice. Representative images of eyeball size and ocular structure from the three strains (**A–C**). Representative confocal images of LRP2/megalin (green) in retinal cryosections (scale bar = 10 μm) (**D**), quantification of its expression (*n* = 4–9 mice per strain) (**E**), and its linear correlation with axial length (*n* = 15 mice) (**F**). Measurements of axial length (**G**), refractive error (**H**), and IOP (**I**) at different time points (*n* = 7–45 mice per strain/age). Quantification of intraocular structure changes (*n* = 7–11 mice per strain) (**J**). *p < 0.05, **p < 0.01, ***p < 0.001, ****p < 0.0001; analyses were performed using one-way ANOVA followed by Bonferroni multiple comparison (**E,J**) and two-way ANOVA followed by Bonferroni multiple comparison (**G–I**). CCT, central corneal thickness; CD, corneal diameter; ACD, anterior chamber depth; LT, lens thickness; VCD, vitreous chamber depth.

Among the 184 CKO mice examined at 2 weeks of age, 85 were female (46.2%) and 99 were male (53.8%). Of these mice, 110 (59.8%) were defined as CKO phenotype^+^, characterized by significantly elongated axial length (AL) compared with age-matched control littermates. The remaining 74 mice (40.2%) were defined as CKO phenotype^−^, i.e., AL was comparable to age-matched controls (Figures 1A–C). Immunofluorescent staining showed that LRP2 knockdown efficiency in RPE cells was positively correlated with AL (Figures 1D–F). These findings support a dose–dependent role of RPE LRP2 in regulating ocular growth and indicate that a threshold level of LRP2 loss may be required for pathological axial elongation to occur. Furthermore, mosaic recombination has been reported previously in Best1-Cre transgenic lines (Iacovelli et al., 2011), supporting the interpretation that variability in recombination efficiency contributes to the observed differences in phenotypic penetrance. CKO phenotype^−^ mice were included in subsequent analyses as a separate subgroup rather than being excluded.

At 2 weeks of age, AL in CKO phenotype^+^ mice (3.58 ± 0.24 mm) was significantly longer than wild-type (WT) mice (2.82 ± 0.06 mm) and CKO phenotype^−^ mice (2.78 ± 0.06 mm). In CKO phenotype^+^ mice, AL progressively increased and reached plateau by 9 months of age (Figure 1G).

Refractive errors progressed to approximately −11.23 D in CKO phenotype^+^ mice at 1 month of age (Figure 1H). No significant intergroup differences in IOP were detected (Figure 1I). By 3 months, the mice exhibited marked ocular remodeling, including increased VCD and CD, decreased ACD and LT, and unchanged CCT (Figure 1J). To reduce inter-individual variability, subsequent experiments were performed using 3-month-old CKO phenotype^+^ mice due to their severe phenotypes.

### 4.2 Retinal thickness and visual function in CKO mice

**Figure 2:**
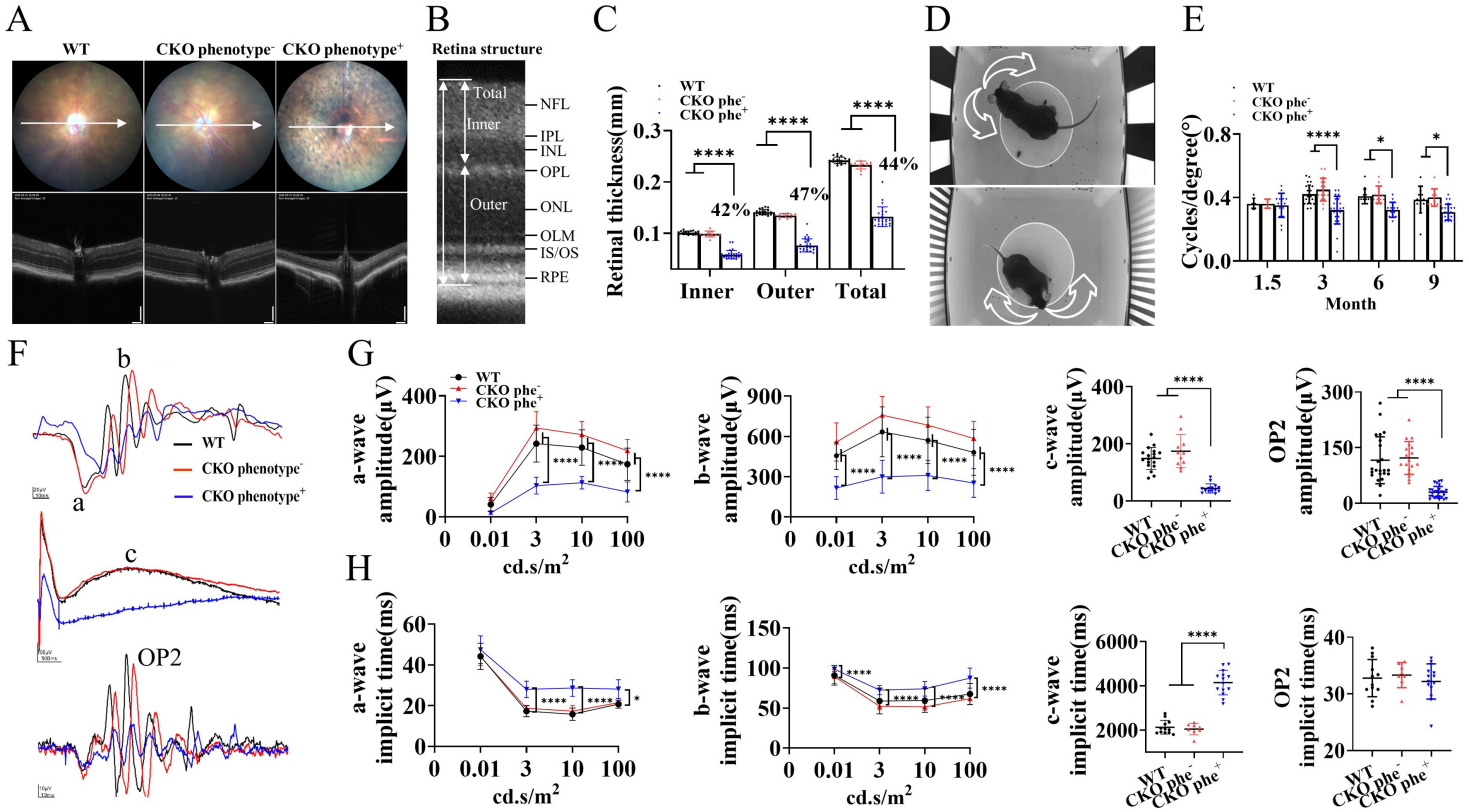
Retinal thickness changes and visual function impairment in 3-month-old CKO mice. Representative fundus and SD-OCT images from the three strains **(A)**, a schematic illustrating retinal thickness measurements **(B)**, and quantitative analysis of retinal thickness (*n* = 9–16 mice per strain) **(C)**. Schematic of the optomotor response test and quantification analysis (**D,E**). Representative scotopic ERG responses obtained from the three strains (**F**). Quantification of the amplitudes (μV) and implicit times (ms) of the a-, b-, and c-wave and OP2 (*n* = 8–24 mice per group) (**G,H**). *p < 0.05, ****p < 0.0001; two-way ANOVA with Bonferroni correction for a-wave, b-wave, and optomotor responses; One-way ANOVA with Bonferroni correction for c-wave and OP2. RPE, retinal pigment epithelium; IS/OS, photoreceptor inner/outer segments; ONL, outer nuclear layer; OPL, outer plexiform layer; INL, inner nuclear layer; IPL, inner plexiform layer; NFL, nerve fiber layer.

OCT revealed generalized retinal thinning and atrophy of the RPE–choroid complex in 3-month-old CKO phenotype^+^ mice (Figures 2A–C), and the pathological changes progressed to 9 months of age (Supplementary file 1 Figure S1).

In line with the retinal structural changes, optomotor responses were significantly impaired in CKO phenotype^+^ mice (Figures 2D,E). Scotopic ERG showed significant reductions in the amplitudes of a-, b-, and c-waves and OP2, accompanied by prolonged implicit times (Figures 2F–H), with no additional decline at 6 or 9 months (Supplementary file 1 Figure S2). These results demonstrate severe visual function impairment in CKO phenotype^+^ mice.

### 4.3 Fundus alterations in CKO mice

At 3 months, CKO phenotype^+^ mice displayed vascular attenuation and retinal pigmentation (100%), accompanied by tilted optic discs (80%) and peripapillary atrophy (75%). Tessellated fundus was observed in 70% of 3-month-old mice and increased to 100% by 6 months of age. At the same time, the prevalence of tilted discs and peripapillary atrophy increased to 85%. By 9-month-old, the prevalence of peripapillary atrophy and choroidal atrophy reached 90% and 70%, respectively (Supplementary file 1 Figure S3).

### 4.4 Retinal and choroidal blood flow in CKO mice

**Figure 3:**
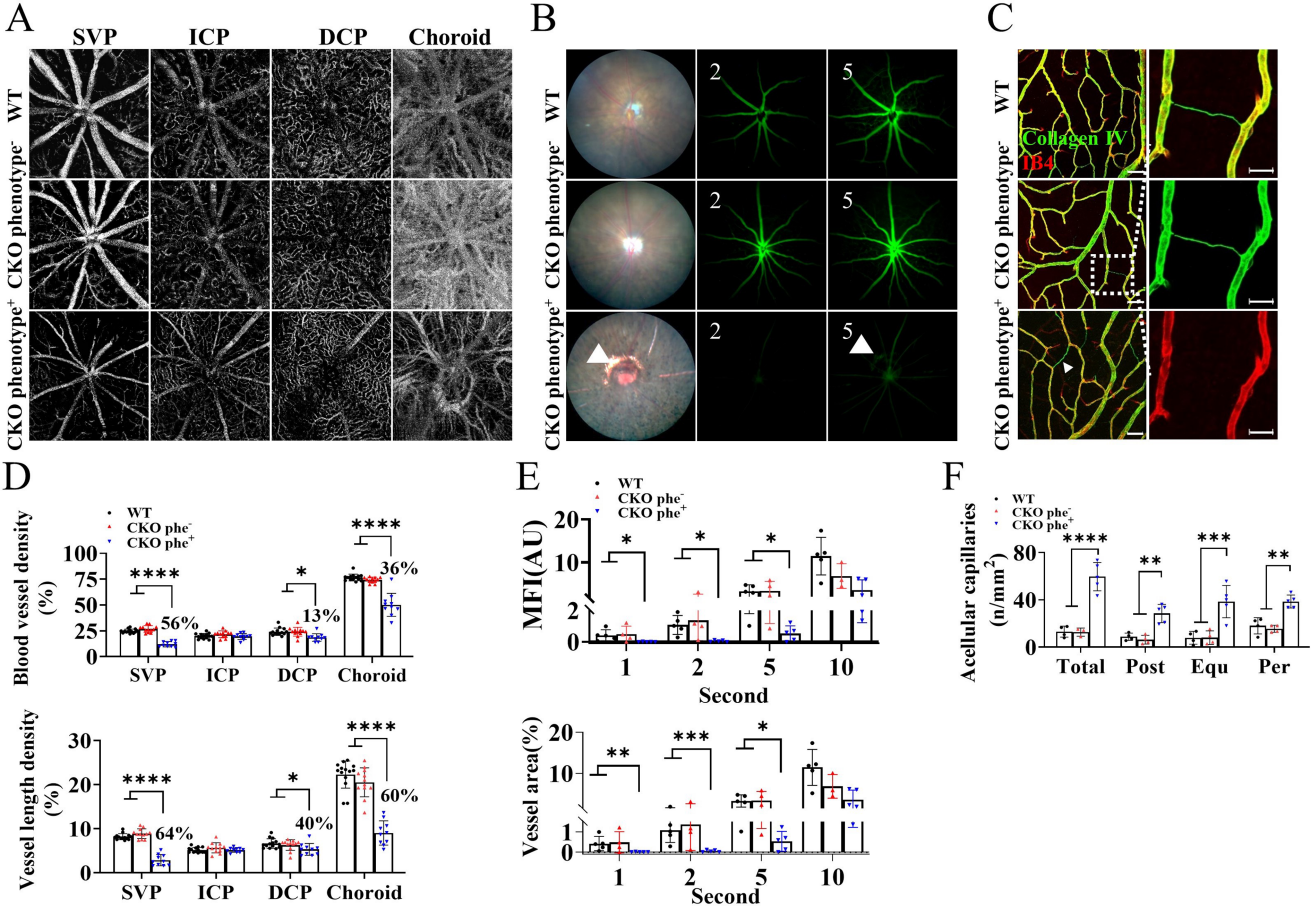
Vascular changes in 3-month-old CKO mice. Representative OCTA images **(A)** and quantification of SVP, ICP, DCP, and choroid **(D)** (*n* = 5–8 mice per group). Representative fundus fluorescein angiography (FFA) images **(B)** and quantification of mean fluorescence intensity (MFI) **(E)** (*n* = 4–5 mice per strain). Representative retinal flat mounts stained for Collagen-IV (red) and IB4 (green) **(C)** and quantification of acellular capillaries **(F)** (*n* = 6–8 mice per strain). Scale bar = 50 μm, peripapillary atrophy (arrow). *p < 0.05, **p < 0.01, ***p < 0.001, ****p < 0.0001; One-way ANOVA followed by Bonferroni multiple comparison. SVP, superficial vascular plexus; ICP, intermediate capillary plexus; DCP, deep capillary plexus; MFI, Mean Fluorescence Intensity; AU, Arbitrary Units.

At 3 months, CKO phenotype^+^ mice exhibited significantly reduced blood flow density and vascular length density in both the retina and choroid (Figures 3A,D). FFA revealed delayed and weakened fluorescence with significantly smaller filling areas and reduced mean fluorescence intensity (MFI) at 1, 2, and 5 min (Figures 3B,E) after sodium fluorescence injection. Retinal flatmount analysis revealed increased density of Collagen-IV^+^/IB4^−^ acellular capillaries (Figures 3C,F).

### 4.5 Retinal cellular alterations in CKO mice

To accurately evaluate AL elongation-induced retinal degeneration at the cellular level, we measured retinal areas in different groups. At 3 months, the retinal length (in cryosections) and area (in flatmount) of CKO phenotype^+^ mice increased by 1.74-fold and 1.98-fold, respectively; whereas retinal thickness and volume reduced by 55.95% and 20.58%, respectively, compared with age-matched WT and CKO phenotype^−^ mice (Supplementary file 1 Figure S4). The results suggest a 20% reduction in the overall retinal volume in CKO phenotype^+^ mice. In the subsequent retinal cell analysis, we adjusted retinal area for absolute cell number calculation.

**Figure 4:**
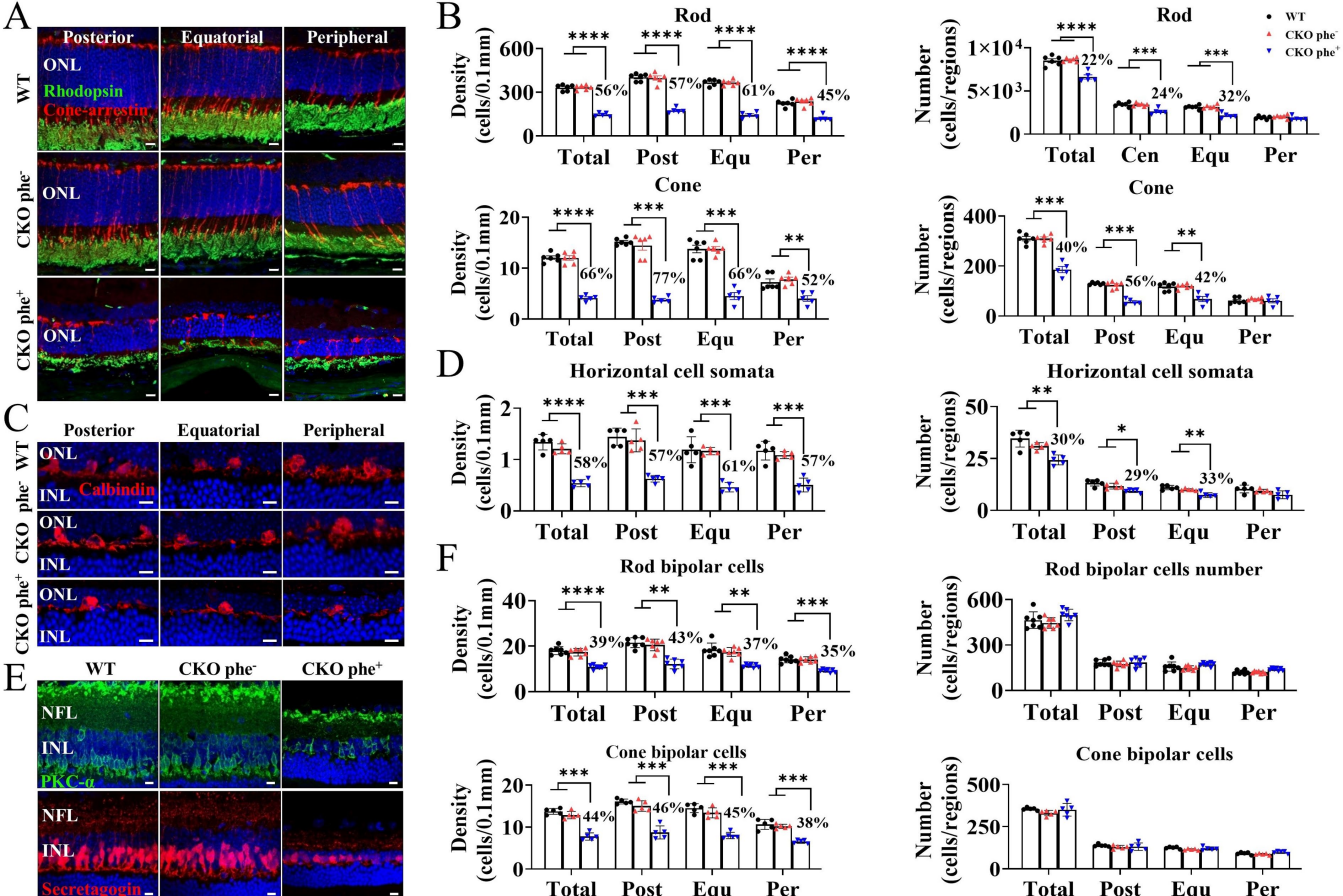
Changes in photoreceptor, horizontal, and bipolar cells in 3-month-old CKO mice. Representative immunofluorescence images and quantification of retinal cryosections stained for cone-arrestin^+^ cone cells (red) and rhodopsin^+^ rod cells (green) **(A,B)**, calbindin^+^ horizontal cells (red) **(C,D)**; PKC-α^+^ rod bipolar cells (green) and SCGN^+^ cone bipolar cells (red) **(E,F)**. Scale bar = 10 μm. *n* = 21–25 retinal images from 5 mice per strain. For quantification, multiple images from the same mouse were averaged to generate one value per animal. *p < 0.05, **p < 0.01, ***p < 0.001, ****p < 0.0001; one-way ANOVA followed by Bonferroni multiple comparison. NFL, nerve fiber layer; IPL, inner plexiform layer; INL, inner nuclear layer; ONL, outer nuclear layer.

Photoreceptor cells: Rod cell density decreased by 56% (57% in posterior, 61% in equatorial, and 45% in peripheral). The total number of rod cells decreased by 22% (24% in posterior and 32% in equatorial, no significant change in peripheral, Figures 4A,B). Cone-arrestin^+^ cell density decreased by 66% (77% in posterior, 66% in equatorial, and 52% in peripheral) in CKO phenotype^+^ mice. The total number of cone cells declined by 40% (56% in posterior and 42% in equatorial, no significant change in the periphery, Figures 4A,B).

Horizontal and bipolar cells: Calbindin^+^ horizontal cell density decreased by 58% (57% in posterior, 61% in equatorial, 57% in peripheral) in CKO phenotype^+^ mice. The total number of horizontal cells declined by 30% (29% in posterior and 33% in equatorial, no significant change in peripheral, Figures 4C,D). PKC-*α* and SCGN were used to identify rod and cone bipolar cells, respectively. In CKO phenotype^+^ mice, rod and cone bipolar cell densities decreased by approximately 39% and 44%, respectively, across all retinal regions, whereas their total and regional cell numbers remained unchanged (Figures 4E,F).

**Figure 5:**
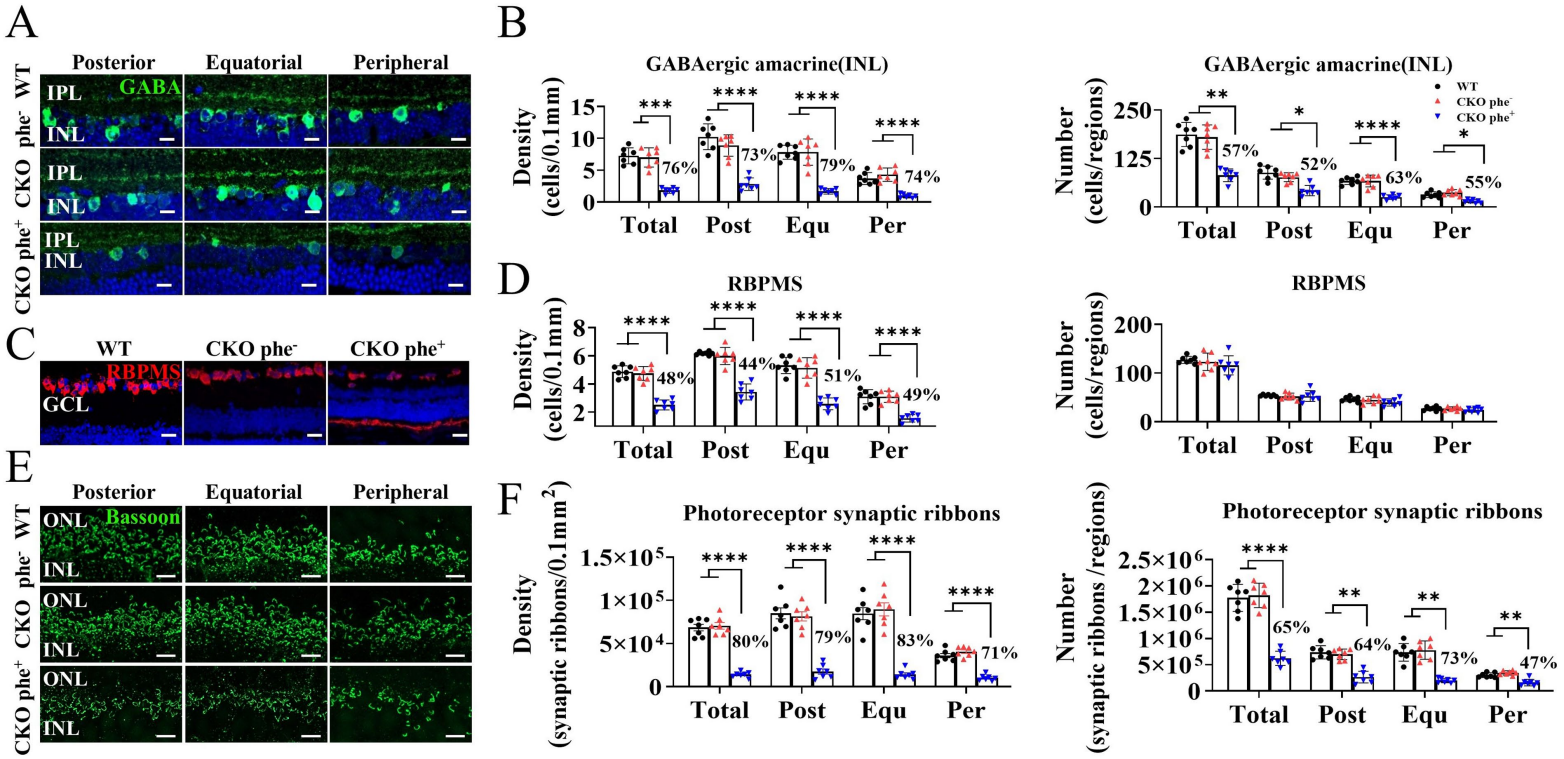
Changes in amacrine cells, ganglion cells and photoreceptor synaptic ribbons in 3-month-old CKO mice. Representative immunofluorescence images of retinal cryosections and corresponding quantification of GABA^+^ amacrine cells (**A,B**; green; scale bar = 10 μm), RBPMS^+^ retinal ganglion cells in the posterior pole region (**C,D**; red; scale bar = 20 μm), and Bassoon^+^ photoreceptor synaptic ribbons (**E,F**; green; scale bar = 5 μm). *n* = 20–26 retinal images from 7 mice per strain. For quantification, multiple images from the same mouse were averaged to generate one value per animal. *p < 0.05, **p < 0.01, ***p < 0.001, ****p < 0.0001; one-way ANOVA with Bonferroni correction. GCL, ganglion cell layer; IPL, inner plexiform layer; INL, inner nuclear layer; ONL, outer nuclear layer.

GABA^+^ amacrine cells and retinal ganglion cells (RGCs): GABA^+^ amacrine cell density decreased by 76% in CKO phenotype^+^ mice. The total number of amacrine cells decreased by 57% (52% in posterior, 63% in equatorial, and 55% in peripheral, Figures 5A,B). Compared with WT and CKO phenotype^−^ mice, the density of RBPMS^+^ RGCs decreased by 48% in CKO phenotype^+^ mice (44% in posterior, 51% in equatorial, and 49% in peripheral). The total number of RGCs did not significantly differ among the three groups (Figures 5C,D), and the regional distribution of RGC counts is schematically illustrated in Supplementary file 1 Figure S5.

Synaptic ribbons: CKO phenotype^+^ mice showed an 80% reduction in Bassoon^+^ synaptic ribbon density (79% in posterior, 83% in equatorial, and 71% in peripheral). The total ribbon number decreased by 65% (64% in posterior, 73% in equatorial, and 47% in peripheral, Figures 5E,F).

In general, our results suggest a cell type (photoreceptors, horizontal cells, and amacrine cells) and regional (posterior and equatorial) specific reduction of retinal neurons in response to AL elongation in myopic eyes.

Next, we investigated how retinal supportive cells responded to neuronal degeneration.

**Figure 6:**
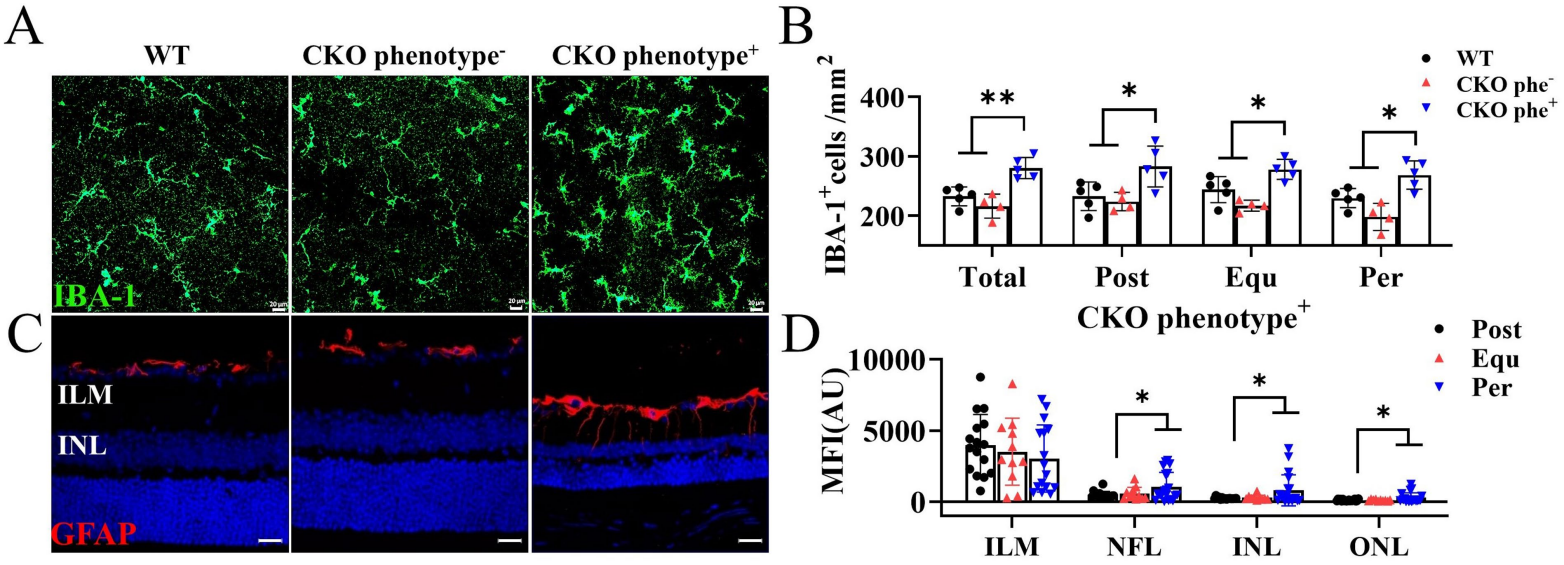
Activation of retinal Müller glia and microglia in 3-month-old CKO mice. Representative retinal flat mounts stained for IBA-1^+^ microglia (green) and quantification of microglial cell numbers (*n* = 4–5 mice per strain), scale bar = 20 μm (**A, B**). Representative retinal cryosections stained for GFAP immunoreactivity (red) and quantification of mean fluorescence intensity (MFI) (*n* = 21–25 images from 5 mice per strain). Scale bar = 20 μm (**C, D**). For image-based quantification, multiple images from the same mouse were averaged to generate one value per animal. *p < 0.05, **p < 0.01; one-way ANOVA followed by Bonferroni multiple comparison. ILM, internal limiting membrane; INL, inner nuclear layer; ONL, outer nuclear layer; MFI, Mean Fluorescence Intensity (Arbitrary Units).

Microglia and Müller cells: CKO phenotype^+^ mice had significantly higher number of IBA-1^+^ microglia in all regions compared to WT and CKO phenotype^−^ mice (Figures 6A,B). Their microglia exhibited enlarged and rounded somas with reduced and shortened dendrites, indicative of mild activation (Figures 6A,B). Notably, we detected significant IBA-1^+^ microglia accumulation in photoreceptor layer, particularly in posterior and equatorial regions, with ameboid morphology (Supplementary file 1 Figure S6). The GFAP^+^ immunoreactivities were restricted in the nerve fiber layer in WT and CKO phenotype^−^ mice (Figures 6A,B), but extended to OPL in CKO phenotype^+^ mice, suggesting significant Müller cell activation (Figures 6C,D).

**Figure 7:**
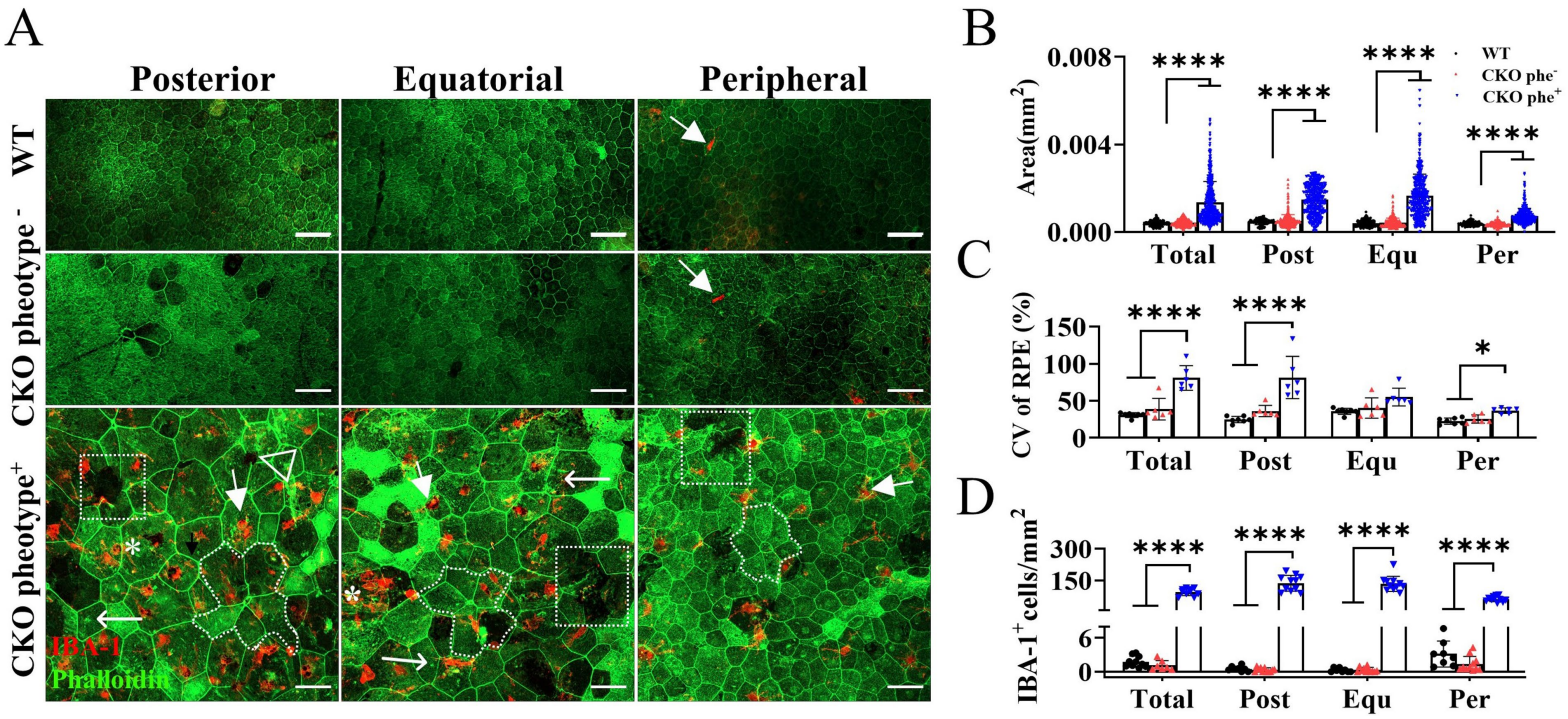
RPE morphological alterations and microglial activation in 3-month-old CKO mice. Representative RPE/choroid flat mounts co-stained with phalloidin (F-actin, green) and IBA-1^+^ microglia (red), scale bar = 50 μm. Microglia (thick arrows), vacuoles (thin arrows), pigment-laden macrophages (triangles), degenerative or hypertrophic RPE cells (rosettes), giant RPE cells (asterisks), and indistinct RPE boundaries suggestive of macrophage infiltration (squares) **(A)**. Quantification of RPE cell area (*n* = 367–525 cells from 6 to 8 mice per strain), coefficient of variation (CV) of RPE cell area (*n* = 6–7 mice per strain), and the number of IBA-1^+^ microglia in the RPE (*n* = 6–7 mice per strain) **(B–D)**. *p < 0.05, ****p < 0.0001; one-way ANOVA followed by Bonferroni multiple comparison.

RPE flatmounts: CKO phenotype^+^ mice displayed significantly enlarged, irregular hexagonal RPE cells with blurred boundaries, scattered pigment granules, and round pigment-containing cells (likely melanin-phagocytosing macrophages). Large RPE cells often contained vacuoles, suggesting cellular damage. These changes were most prominently in posterior and equatorial regions (Figures 7A,C). The RPE cell area and coefficient of variation (CV) were significantly higher in CKO phenotype^+^ mice, particularly in the posterior region, compared with controls (Figure 7C).

CKO phenotype^+^ mice showed numerous IBA-1^+^ activated microglia in the subretinal space, with higher densities in posterior and equatorial regions than in the periphery and containing internalized pigment granules consistent with phagocytosed RPE debris. IBA-1^+^ cells were occasionally detected in the peripheral area in WT and CKO phenotype^−^ mice (Figure 7D).

## 5 Discussion

Pathological myopia is increasingly recognized as a complex neurodegenerative disorder involving coordinated changes in ocular growth, retinal neurons, vasculature, and supporting tissues. In this study, we demonstrate that RPE-specific depletion of Lrp2 leads to early-onset and progressive ocular enlargement accompanied by retinal vascular, neuronal, and choroidal degeneration, closely recapitulating key features of human myopic retinopathy. While enlarged eyeballs and retinal thinning have been reported previously in Lrp2-deficient models (Cases et al., 2015), our study reveals a previously unrecognized cell type-specific pattern of retinal neurodegeneration (rather than uniform neuronal loss), providing important mechanistic insights into myopia-associated vision loss.

A striking finding is that despite a two-fold expansion of the total retinal area by 3 months of age, retinal thickness is markedly reduced, indicating tissue stretching and structural decompensation rather than proportional growth. Importantly, this enlargement is accompanied by retinal vascular and neuronal degeneration. Interestingly, the true loss of neurons is restricted to specific subtypes, including GABAergic amacrine cells, horizontal cells, photoreceptors and their synaptic ribbons, whereas RGCs and bipolar cells exhibit reduced density without a reduction in absolute cell number. This distinction is critical, as it suggests that axial elongation does not uniformly induce retinal neuron loss, but instead selectively compromises neuron types. Since different neurons have different capacities to deal with mechanical stress, metabolic demand, or microenvironmental disruption (Tan et al., 2006; Liu and Prokosch, 2021; Jackson et al., 2024), the progressive vascular degeneration would disproportionately affect metabolically demanding neurons, thereby contributing to the selective loss observed in CKO mice. Further studies will be needed to establish the causal link between vascular degeneration and selective retinal neuronal loss.

Photoreceptors and horizontal cells reside in the outer retina, where their survival and function depend critically on intact RPE support and adequate retinal and choroidal perfusion (Strauss, 2005; Elsner et al., 2020). In the CKO mice, extensive RPE dysmorphology and patchy choroidal atrophy are expected to disrupt the metabolic and homeostatic interactions between RPE and photoreceptors, including nutrient support, retinoid recycling, and ion regulation (Kurihara et al., 2016; Kanow et al., 2017; Swarup et al., 2019; Chen et al., 2024; Hansman et al., 2025). Given their exceptionally high metabolic demand, photoreceptors are particularly vulnerable to impairments in energy supply and tissue homeostasis (Guadagni et al., 2015; Fu et al., 2019; Hass et al., 2023), making them a likely target of RPE and choroidal dysfunction. Horizontal cells may also be especially susceptible to alterations in the outer retinal microenvironment. As the first-order interneurons receiving direct photoreceptor input, they rely on continuous synaptic communication with photoreceptors and participate in complex neurotransmitter-mediated circuits involved in visual adaptation and refractive development (Negishi and Drujan, 1979; Knapp and Dowling, 1987; Lu and McMahon, 1997; Weiler et al., 1998; McMahon and Schmidt, 1999; Weiler et al., 2000; Sun et al., 2009). Notably, horizontal cells appear relatively resistant to mechanical stress induced by elevated intraocular pressure (Chun et al., 1999). Their extensive gap junction coupling and marked dendritic plasticity further suggest a high sensitivity to local metabolic and physiological changes (Ajioka et al., 2007). Their degeneration in the present model is more likely related to disruption of the outer retinal environment than to retinal stretching per se. Taken together, these observations suggest that RPE dysfunction and associated choroidal abnormalities may contribute to the selective loss of photoreceptors and horizontal cells in our CKO mice. Nevertheless, a direct causal relationship between RPE dysfunction and outer retinal degeneration was not established in the current study and will require further investigation.

The degeneration of amacrine cells is of particular relevance to myopia progression (Zhang et al., 2024) since they are critically involved in shaping retinal circuitry, modulating contrast sensitivity, and regulating retinal dopamine signaling (Tien et al., 2016; Banerjee et al., 2020). Dopaminergic amacrine cells, in particular, have been implicated in the control of eye growth and refractive development, with reduced retinal dopamine signaling consistently associated with experimental myopia (Ling et al., 2024; Mehler et al., 2025). Although our study focused on GABAergic amacrine populations, their loss may disrupt inhibitory networks and indirectly alter dopaminergic signaling, thereby exacerbating dysregulated eye growth and promoting a feed-forward cycle of axial elongation and retinal degeneration. The GABAergic amacrine cells provide lateral and temporal inhibition within the inner retina, sculpting and refining the visual signal before it is sent to the brain (Masland, 2012; Zhang and McCall, 2012; Diamond, 2017). GABAergic neurotransmission is tightly coupled to cellular energy metabolism via the astrocyte-dependent TCA cycle, and is particularly sensitive to disturbances in the metabolic microenvironment (Occhipinti et al., 2010; Calvetti and Somersalo, 2012). GABAergic amacrine cells are vulnerable to chronic mechanical stress (Akopian et al., 2019). The selective loss of GABAergic amacrine cells suggests that inhibitory interneurons may be particularly sensitive to retinal stretching or altered extracellular signaling during eye enlargement, potentially contributing to early functional deficits before widespread neuron loss.

Furthermore, amacrine cells are increasingly recognized as important modulators of neurovascular coupling in the retina (Usui et al., 2015; Midena et al., 2021). Loss of inhibitory amacrine input could impair activity-dependent regulation of retinal blood flow, contributing to vascular instability and degeneration. Thus, degeneration of amacrine cells in the CKO retina may not only be a consequence of myopic pathology, but also an active contributor to disease progression by amplifying vascular dysfunction and retinal metabolic stress.

In contrast, the preservation of absolute numbers of RGCs and bipolar cells, despite reduced density, suggests that these neurons tolerate retinal stretching and reduced vascular support, redistributing over an expanded retinal area rather than undergoing degeneration. This observation parallels clinical findings in myopic patients, where retinal thinning and reduced cell density are common, yet substantial neuronal loss, particularly of RGCs, often occurs later or in association with secondary pathologies such as glaucoma or ischemia (Stapley et al., 2020, 2023). Our data therefore support a model in which axial elongation initially leads to retinal “dilution,” followed by selective degeneration of vulnerable neuronal populations as structural and metabolic stress exceeds compensatory capacity.

The upregulation of Müller cell GFAP and pronounced microglial activation observed in the CKO retina underscore the contribution of neuroinflammation to myopic retinopathy. Previous studies have demonstrated elevated levels of inflammatory mediators, such as IL-6 and MMP-2 in highly myopic eyes, which positively correlate with axial length and indicate the presence of chronic low-grade intraocular inflammation (Yuan et al., 2019; Yang et al., 2025). Furthermore, higher intraocular pro-inflammatory cytokines, including Chi3l1, IL-6Rα and IL-8, have been reported to be associated with retinal vascular impairment and neuronal degeneration in myopic retinopathy (Zeng et al., 2024). The accumulation of IBA-1^+^ cells in the subretinal space, together with RPE disruption, suggests that retinal immune privilege is compromised in myopic eyes.

This study has several limitations. First, the eyeball size continues grows from 2 weeks to 9 months of age in CKO phenotype^+^ mice, but retinal degenerative changes, including the neurons, vasculature, and RPE cells, were assessed at a single time point, i.e., three-month-old mice. We do not know which of the components are most sensitive to mechanical stretch-related stress in pathological myopia. Second, we used retinal section, not flatmount, to assess the number and density of retinal neurons. As shown in our study, the density of some retinal neurons such as RGCs and amacrine cells differ significantly in different retinal regions. This approach reduced data accuracy, particularly for the non-uniformed cells. Third, the study did not address the causal link between retinal vascular, neuronal, and RPE degeneration. Further studies will be needed to address these limitations.

## 6 Conclusion

Our results suggest that the CKO mice mirror closely many features of retinal pathology seeing in patients with pathological myopia. Using this model, we identify selective neuronal degeneration, not merely reduced cell density, as a defining feature of myopic retinopathy. Our work highlights amacrine cells, retinal vasculature, and RPE as critical, yet underappreciated, contributors to myopic retinopathy and suggests that effective therapeutic strategies should aim to preserve neurovascular integrity in addition to controlling axial elongation.

## Data Availability

The original contributions presented in the study are included in the article/Supplementary material, further inquiries can be directed to the corresponding author.
