# Supplementary material for: Selective retinal neuron loss and impaired neurovascular support underlie myopic retinopathy in RPE-specific Lrp2-deficient mice
Source: Front Cell Neurosci. 2026 Jul 30;20:1856140. doi: 10.3389/fncel.2026.1856140 (PMC13467757; doi:10.3389/fncel.2026.1856140)
Supplement: Supplementary file 1 [file Supplementary_file_1.DOCX]

**Table S1 Primer sequences for mouse genotyping.**

| Primer gene | Forward sequence 5'-3' | Reverse sequence 5'-3' |
| --- | --- | --- |
| *Lrp2* | TGTAAGGGACGTTCAGCCTTTTA | TGCTGAATGCAAGAAAAGTGAAGC |
| *Best1-Cre* | ATGCCCAAGAAGAAGAGGAAG | TGGCCCAAATGTTGCTGGATAGTTTTTA |

**Table S2** **Antibodies used in the study**

| Antigen | ID | Dilution | Source |
| --- | --- | --- | --- |
| Rabbit polyclonal anti-Lrp2 | ab76969 | 1:300 | Abcam |
| Rabbit polyclonal anti-Collagen IV | ab19808 | 1:300 | Abcam |
| Griffonia Simplicifolia IB4 | B-1205-.5 | 1:100 | Vectorlabs |
| Mouse monoclonal anti-Rhodopsin | ZMS1093 | 1:10000 | Chemicon |
| Rabbit polyclonal anti-Cone arrestin | AB15282 | 1:10000 | Chemicon |
| Mouse monoclonal anti-Bassoon | ADI-VAM-PS003-F | 1:400 | Enzo |
| Rabbit polyclonal anti-SCGN | PA5-30393 | 1:250 | Invitrogen |
| Rabbit polyclonal anti-Calbindin | PA5-85669 | 1:250 | Invitrogen |
| Rabbit monoclonal anti-PKCα | ab32376 | 1:300 | Abcam |
| Rabbit polyclonal anti-GABA | PA5-32241 | 1:400 | Invitrogen |
| Rabbit polyclonal anti-RBPMS | 151187-1-AP | 1:300 | Proteintech |
| Rabbit monoclonal anti-Iba-1 | 012-26723 | 1:400 | Wake |
| Rat monoclonal anti-GFAP | 13-0300 | 1:200 | Therom |
| Phalloidin-iFluor 488 | ab176753 | 1:1000 | Abcam |
| Alexa Fluor® 488 goat anti-mouse IgG | A-11001 | 1:500 | Invitrogen |
| Alexa Fluor® 594 goat anti-mouse IgG | A-11005 | 1:500 | Invitrogen |
| Alexa Fluor® 488 goat anti-rat IgG | A-11006 | 1:500 | Invitrogen |
| Alexa Fluor® 594 goat anti-rat IgG | A-11007 | 1:500 | Invitrogen |
| Alexa Fluor® 488 goat anti-rabbit IgG | A-11008 | 1:500 | Invitrogen |
| Alexa Fluor® 594 goat anti-rabbit IgG | A-32740 | 1:500 | Invitrogen |

**Figure S1.** **Measurements retinal thickness at different time points from three strains**. n = 12–39 mice per strain. **p* < 0.05, ***p* < 0.01, ****p* < 0.001, *****p* < 0.0001, one-way ANOVA followed by Bonferroni multiple comparison.

**Figure S2.** **Quantification of the amplitudes (μV) and implicit times (ms) of the scotopic a-, b-, c-wave and OP2 at 3, 6 and 9 months of age**. N = 13–27 mice per group, **p* < 0.05, ***p* < 0.01, ****p* < 0.001, *****p* < 0.0001. Two-way ANOVA followed by Bonferroni multiple comparison.

**Figure S3. Retinal degeneration changes in the CKO mice.** Myopic tilted disc (vertical frame), peripapillary atrophy (square), retinoschisis (arrow), vitreous traction (triangle), chorioretinal atrophy (star).

**Figure S4.** **Changes of retinal length and area in 3-month-old CKO mice**. Representative immunofluorescence images of retinal cryosections (DAPI, blue, scale bars = 200 µm) and retinal flat mount (Collagen IV, red, scale bars = 500 or 1000 µm) (**A**). Quantification of retinal length, area, thickness and volume, n = 5–8 mice per strain **(B–D)**, ****p* < 0.001, *****p* < 0.0001, one-way ANOVA followed by Bonferroni multiple comparison.

**Figure S5. Schematic of regional RGC counts distribution.**

**Figure S6.** **Activation and migration of retinal microglia in 3-month-old CKO mice**. Representative retinal flat-mount images showing IBA-1^+^ microglia (green) in the ganglion cell layer (GCL), inner plexiform layer (IPL), outer plexiform layer (OPL), and photoreceptor layer (PRL), scale bar = 20 µm (**A**). Quantitation of microglial cell numbers, n = 20–24 retinal images from 4–6 mice per strain (**B**). **p* < 0.05, one-way ANOVA followed by Bonferroni multiple comparison.
